# Exosomes, autophagy, and cancer: A complex triad

**DOI:** 10.1002/ijc.35388

**Published:** 2025-05-02

**Authors:** María Guerra‐Andrés, Álvaro F. Fernández, Tania Fontanil

**Affiliations:** ^1^ Departamento de Bioquímica y Biología Molecular Universidad de Oviedo Oviedo Spain; ^2^ Instituto Universitario de Oncología del Principado de Asturias (IUOPA) Oviedo Spain; ^3^ Instituto de Investigación Sanitaria del Principado de Asturias (ISPA) Oviedo Spain; ^4^ Instituto Ordoñez (Astracime S.L) Oviedo Spain; ^5^ Lovinium Biocell CO LTD. Bangkok Thailand

**Keywords:** angiogenesis, autophagy, cancer, exosomes, metastasis, tumor

## Abstract

Cancer remains one of the leading causes of death worldwide. Despite remarkable progress in prevention, diagnosis, and therapy, the incidence of certain types of cancer persists, urging the identification of clinically relevant biomarkers and the development of novel therapeutic strategies to improve clinical outcomes and overcome treatment resistance. Exosomes, small extracellular vesicles released by diverse types of cells, have attracted interest in biomedical research due to their potential as carriers for different treatments. Moreover, exosomes play a pivotal role in intercellular communication, modulating various cellular processes. One of those is autophagy, a pro‐survival pathway that is essential for human cells. Even though autophagy is traditionally described as a catabolic route, its machinery is intricately involved in various cellular responses, including vesicle formation and secretion. In this regard, the link between autophagy and exosomes is complex, bidirectional, and highly dependent on the cellular context. Interestingly, both processes have been extensively implicated in cancer pathogenesis, highlighting their potential as therapeutic targets. This review updates our understanding of how exosomes can participate in cancer development and progression, with a specific focus on their influence on tumor growth, angiogenesis, and metastasis. Additionally, the interplay between these extracellular vesicles and autophagy is minutely reviewed and discussed, as we hypothesize that this crosstalk may hold valuable clues for biomarker discovery and the development of novel therapeutic strategies.

## Graphical abstract

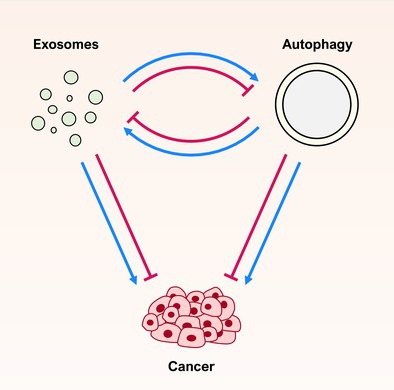

## 1 INTRODUCTION

**FIGURE 1:**
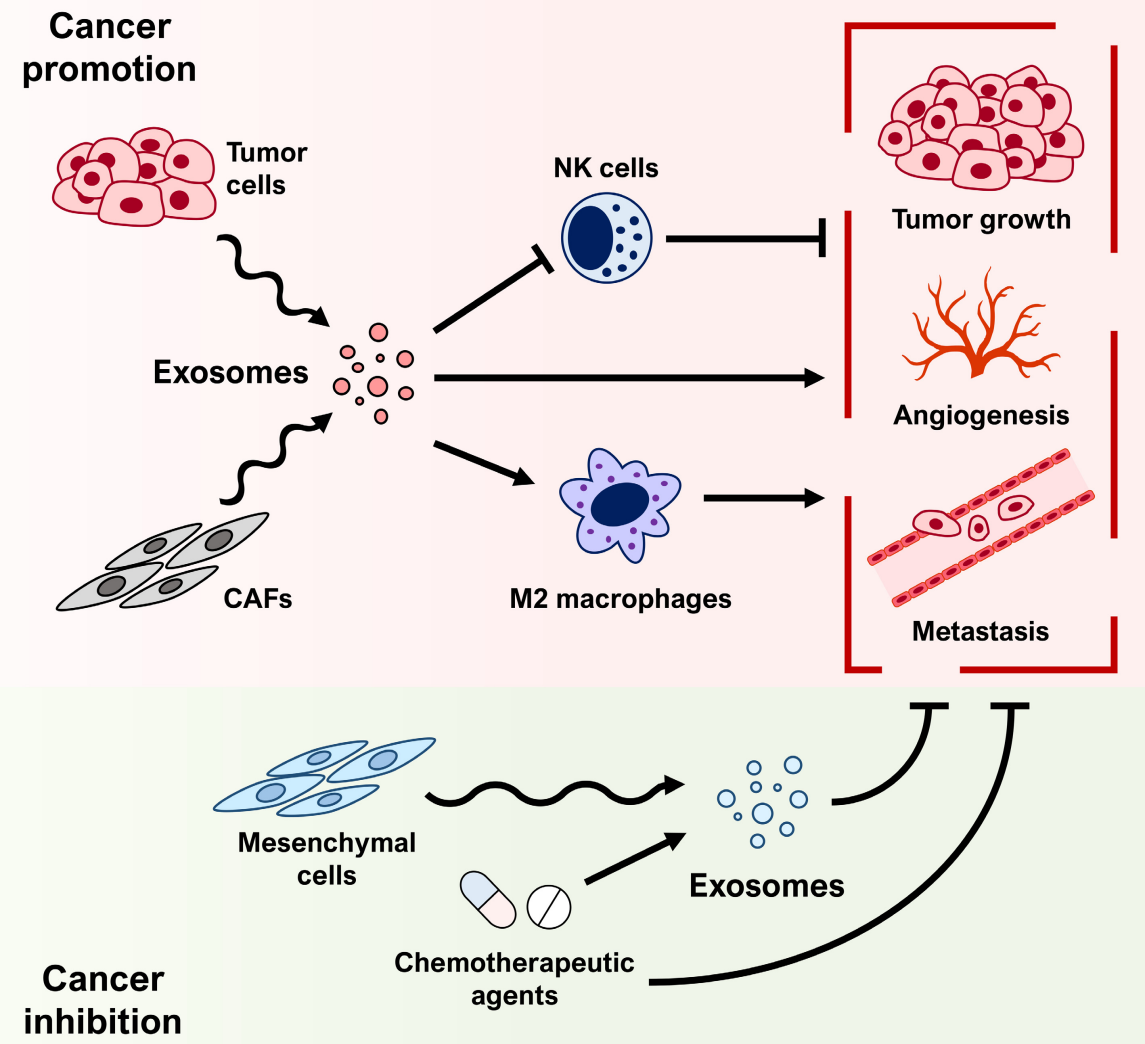
Exosomes and cancer. Diagram summarizing the effects of exosomes on tumors and the tumor microenvironment, depending on their different origins. Tumor cells and cancer‐associated fibroblasts (CAFs) produce exosomes that can promote the progression of the disease, also by hindering the action of NK cells or inducing M2 polarization in macrophages. Meanwhile, mesenchymal cells or the treatment with chemotherapeutic agents trigger the release of exosomes that hamper tumor growth, angiogenesis, or metastasis.

Cancer is one of the leading causes of mortality worldwide. Just in 2020 (the last year with available data), over 19 million individuals were diagnosed with this disease, and approximately 10 million deaths were recorded.^1^ Despite the prevalence of cases surpassing 50 million, it is encouraging to observe that the mortality rate is declining, thanks to increasing efforts in prevention, diagnosis, and therapy. However, these advances are still insufficient to counteract the growing number of cases. Therefore, expanding our knowledge of this pathology and our capacity to design new therapies is imperative. In this regard, exosomes have generated considerable interest in the scientific community due to their properties, especially in the fields of medicine and biotechnology.^2^ These extracellular vesicles, approximately 100 nm in size, mediate the transport of different biomolecules, including proteins, lipids, mRNA, and miRNA, from one cell to others, acting as intercellular messengers.^3^, ^4^ This role makes them promising candidates for therapeutic applications in cancer, being able to function as agents that alter the tumor microenvironment or as carriers for various treatments, like chemotherapy.^5^, ^6^, ^7^ It is important to note that the term “exosome” has frequently been negligently used in the literature to describe any population of extracellular vesicles (EV), even mixed ones, even though it refers to a very specific subset of them. In fact, preparations often include other types of small EVs (like “ectosomes”, directly released from the plasma membrane) and non‐vesicular extracellular nanoparticles (NVEPs, including “exomeres” and “supermeres”), as shown by recent studies.^8^, ^9^, ^10^ Additionally, extracellular RNA (exRNA) is not only transported by exosomes but also by other carriers such as the aforementioned NVEPs.^10^, ^11^, ^12^ Therefore, some effects traditionally attributed to exosomes may be due to the combined action of multiple carriers, and thus caution must be adopted when consulting the existing literature. In any case, exosomes play a crucial role in cell communication, modulating various processes. Autophagy, an evolutionarily well‐conserved pathway, is one of them. This recycling mechanism is essential for cell survival, mediating a diverse collection of responses (including membrane reorganization or secretory processes) against different stress stimuli.^13^, ^14^, ^15^ Accordingly, autophagy dysregulation has been linked to a wide range of diseases, from aging‐related pathologies or cardiovascular alterations to neurodegeneration or cancer.^16^, ^17^ Unfortunately, most attempts to translate our increasing knowledge of autophagy to the clinic have not been successful, as autophagy regulation is complex and dynamically changes during the progression of the disease. Thus, it is necessary to clearly define when autophagy needs to be repressed or activated and find novel regulatory mechanisms to achieve fine modulation of this pathway. Here, we give an update on the role of exosomes in cancer, focusing on their effect on tumor growth, angiogenesis, and metastasis (Figure 1) and the interplay between these extracellular vesicles and autophagy, as we hypothesize that this crosstalk may become of interest in the development of new therapeutic strategies.

## 2 EXOSOMES IN TUMOR PROGRESSION

Exosomes actively participate in intricate, communicative networks between cancer and normal cells, inducing significant changes in the latter. Specifically, tumor cells can utilize exosomes to remodel the extracellular matrix, create microenvironments favorable to their growth, facilitate invasion, and evade apoptosis, thereby promoting tumor proliferation.^18^, ^19^ In the context of renal cancer, for example, exosomes from tumor cells lacking the Von Hippel–Lindau (*VHL*) gene, a well‐known tumor suppressor,^20^ can transform normal cells, significantly increasing invasion and migration.^21^ However, exosomes primarily regulate cancer progression through their content in non‐coding RNAs (ncRNA), crucial biological regulators that can be delivered to recipient cells by these extracellular vesicles.^22^, ^23^ In this regard, circular RNAs (circRNA) represent a novel class of endogenous non‐coding RNAs that play a regulatory role in cancer biology by possessing specific binding sites for microRNAs (miRNA).^24^ In hepatocellular tumors, for example, co‐culturing hepatocarcinoma cells with exosomes from mesenchymal cells overexpressing *circ563* significantly impacts cell proliferation and metastasis by altering the expression of *miR‐148a‐3p* and *MTF‐1*.^25^ Interestingly, overexpression of *miR‐148a‐3p* or *MTF‐1* depletion counteracts the tumor effects induced by *circ563*, while exosomes from mesenchymal cells not overexpressing *circ563* decrease cell proliferation and metastasis. These findings were supported by xenograft experiments in nude mice, demonstrating that *circ563*‐enriched exosomes facilitated tumor growth by positively regulating *MTF‐1* expression.^25^ Similar crosstalk between exosomes and cancer progression has been observed in gastric cancer (GC).^26^ For example, cancer‐associated fibroblasts can deliver circRNAs like *circ_0088300*, *circFCHO2*, or *circNEK9* to tumor cells via exosomes, promoting proliferation, invasion, and angiogenesis by targeting *miRNA‐1305*,^27^ *miRNA‐194‐5p*,^28^ and *miR‐409‐3p*,^29^ respectively. Another example of exosome‐mediated regulation in cancer occurs in breast tumors, where exosomes released by cancer cells function as both paracrine and autocrine agents.^30^ This is the case of *miR‐155*, which is present in tumor exosomes and contributes to cancer‐associated cachexia by reprogramming metabolism, regulating the transcription of key proteins such as APC, GSK3β, and PPP1CA (all within the canonical Wnt signaling pathway)^31^ and participating in chemotherapy resistance.^32^ Additionally, enrichment in *miR‐9* and *miR‐155* is observed in exosomes produced by highly metastatic triple‐negative breast cancer cells, targeting *PTEN* and *DUSP14* tumor suppressor genes.^33^ Other exosomal miRNAs, such as *miR‐760*,^34^ *miR‐10b*,^35^ *miR‐222*,^36^ or *miR‐1910‐3p*,^37^ impact the proliferation, invasion, and chemotherapy resistance of breast cancer cells through various signaling pathways including PI3K/AKT or NF‐κB. Meanwhile, exposing bone marrow stromal cells to exosomes from chronic myeloid leukaemia cells loaded with *miR‐320* results in the inhibition of the β‐catenin signaling pathway, leading to the suppression of osteogenesis in bone marrow stromal cells and the remodeling of the cellular microenvironment.^38^

However, it has also been observed that exosomes can exert an opposite impact on cancer, showing anti‐tumoral properties. Low levels of *circ‐ITCH*, for example, have been described in serum exosomes of gastric cancer (GC) patients, and functional assays revealed that the overexpression of *circ‐ITCH* not only inhibited the epithelial‐mesenchymal transition (EMT) but also the proliferation, migration, and invasion of GC cells.^39^ This phenomenon is believed to occur because *circ‐ITCH* sequesters *miRNA‐199a‐5p*, thereby increasing the expression of its target, *Klotho*, an anti‐aging factor with a tumor‐suppressive capacity.^40^ In breast cancer, exosomal miRNAs (like *miR‐134‐5p* ^41^ or *miR‐7‐5p* ^42^) can also hamper tumor progression, and those originating from mesenchymal cells can repress genitourinary neoplasms.^43^ In ovarian cancer, exosomal miRNAs like *hsa‐miR‐124‐3p* ^44^ or *miR‐146a* ^45^ can reduce cell division and enhance the effectiveness of chemotherapeutic drugs.

## 3 EXOSOMES AS REGULATORS OF ANGIOGENESIS

Angiogenesis is a biological process involving the formation of new capillaries from pre‐existing blood vessels, as a direct response to tissue‐specific needs.^46^ Chronic and sustained angiogenesis, a distinctive feature of cancer, plays a vital role in tumorigenesis, supporting the continuous growth of the tumor by providing oxygen and nutrients while removing cellular waste. Once again, the release of exosomes by tumor cells plays a significant role in this process,^47^ as scientific evidence shows that these extracellular vesicles can significantly enhance angiogenesis, highlighting the interplay between cell communication and vascular development in cancer.^48^, ^49^, ^50^ A study conducted in 2023 by Zheng and collaborators revealed that esophageal squamous cell carcinoma (ESCC)‐derived exosomes show significantly increased levels of *miR‐21*, a well‐known regulatory molecule in angiogenesis. Mechanistically, this effect is believed to result from the *miR‐21*‐mediated inhibition of *PTEN*,^51^ which results in the upregulation of the PI3K/AKT signaling pathway. Another prominent example of angiogenesis enhancement by exosomes is observed in non‐small cell lung cancer (NSCLC). When SYT7, a key regulator of exocytosis,^52^ is overexpressed, CEP55‐enriched exosomes are released.^53^ This study suggests that CEP55, previously associated with an unfavorable prognosis in NSCLC patients,^54^ may drive angiogenesis by activating HIF1α, a crucial transcription factor associated with angiogenesis through the AKT/mTOR signaling pathway.^55^ Another negative regulator of tumor angiogenesis, *FOXO1*,^56^ is targeted by microRNAs delivered by exosomes in several types of cancer. In the bladder, for example, *FOXO1* is the target of *miR‐1247‐3p*,^57^ while *miR‐135b‐5p* (present in exosomes released by cancer‐associated fibroblasts) and *miR‐183‐5p* (in exosomes produced by tumor cells) repress *FOXO1* in colon cancer.^58^, ^59^

Although exosomes have been identified as pro‐angiogenic mediators, it is important to note that they can also negatively affect angiogenesis in specific contexts. In this regard, serum from hepatocellular carcinoma patients shows reduced levels of exosomes containing *miRNA‐23a‐5p*, which can inhibit cell proliferation and angiogenesis by targeting peroxiredoxin‐2 (*PRDX2*),^60^ a protein linked to this pathology.^61^ Exosomes loaded with ANGTPL1, a tumor suppressor that can be downregulated in different types of cancer like thyroid, colon, or breast,^62^, ^63^, ^64^ can inhibit angiogenesis in glioblastoma by reducing the expression of VEGFA and blocking the VEGFR2/AKT/eNOS pathway.^65^ Interestingly, the release and content of exosomes can be altered by exposure to chemotherapeutic agents. In breast cancer, tamoxifen treatment causes an increment of *miR‐573* and a reduction of CD146 and VEGF in exosomes, leading to reduced angiogenesis,^66^ though tamoxifen resistance can also be expanded by exosome‐mediated delivery of *miR‐221/222* that blocks the expression of P27 and ERα.^67^ Similarly, docosahexaenoic acid (DHA) treatment causes an increase in exosome secretion and modifications in their content, elevating the levels of *let‐7a, miR‐23b*, *miR‐27a/b*, and *miR‐320b*, all known for their antitumoral and/or antiangiogenic properties.^68^

## 4 EXOSOMES AS METASTASIS MEDIATORS

Given their role in intracellular communication, as well as their already‐mentioned participation in tumor growth and angiogenesis, it is not surprising that exosomes can also mediate metastasis.^69^ In fact, they can intervene at various levels during cancer spread, from creating a favorable premetastatic niche to facilitating immune evasion and promoting the survival of cancer cells circulating in the bloodstream.^70^, ^71^, ^72^, ^73^, ^74^ It has been recently described that exosomes can promote epithelial‐mesenchymal transition (EMT),^75^, ^76^ a fundamental process in metastasis during which epithelial cells acquire mesenchymal cell‐like characteristics, exhibiting properties akin to stem cells and increasing their mobility and invasive capacity.^77^ In gastric cancer, for example, exosomes contain elevated levels of *circNRIP1*, which promotes pulmonary and peritoneal metastasis through EMT by blocking *miR‐149‐5p* and activating the AKT/mTOR axis.^78^ Similarly, the presence of *circUBE2Q2* in exosomes released by gastric cancer cells regulates EMT by repressing *miR‐370‐3p* and activating the STAT3 signaling pathway, resulting in peritoneal and hepatic lymph node metastasis in mice.^79^ Furthermore, in gastric adenocarcinoma, *circ_0038138* delivery by exosomes induces proliferation, migration, invasion, glycolytic activity, and lung metastasis by regulating the *miR‐198/EZH2* axis.^80^ In parallel to EMT, exosomes can also contribute to metastasis by modulating the polarization of tumor‐associated macrophages (TAM), which play a crucial role in the tumor's immunological microenvironment.^81^ These macrophages are primarily classified into two phenotypes, M1 and M2, with opposing functions: while M1 macrophages generate immunostimulatory factors and exert antitumor effects, M2 macrophages promote immunosuppression, sustained tumor growth, angiogenesis, and metastasis. This functional differentiation is mediated by numerous factors such as cytokines, chemokines, signaling pathways, and intercellular interactions.^82^, ^83^, ^84^ In lung cancer, exosomes loaded with circRNA *circ_0001715* and lncRNA *HOXC‐AS2* enhance proliferation and metastasis by inducing the polarization of macrophages toward the M2 phenotype targeting the *miR‐205‐5p/TREM2* axis^85^ and the STAT1/SOCS1 and STAT1/CIITA pathways,^86^ respectively, repressing the former and positively regulating the latter.

Another way exosomes modulate metastasis is by enhancing the immune escape of tumor cells by influencing natural killer (NK) cells,^87^ a key immunological component with remarkable tumor‐killing capacity.^88^ These cytotoxic abilities are largely determined by the activity of their surface receptors, including both activating receptors (such as NKp30, NKp44, NKp46, or NKG2D) and inhibitory receptors (like KIR, PD‐1, NKG2A, or TIM‐3).^89^ The expression of these surface receptors can be affected by exosomes released by tumor cells.^90^, ^91^ For example, it has been demonstrated that oral carcinoma cells deliver extracellular vesicles rich in TGF‐β to NK cells, resulting in cell dysfunction.^92^ Similarly, exosomes derived from melanoma express TGF‐β and inhibit the expression of NKG2D, playing a crucial role in the immunosuppression of NK cells.^93^ Other studies show that exosomes from hepatocellular carcinoma have elevated levels of *circUHRF1*, which inhibits the expression of *miRNA‐449c‐5p* in NK cells, thus increasing the expression of the inhibitory NK cell receptor TIM‐3 and weakening the secretion of antitumor factors such as TNFα and IFN‐γ.^94^ Meanwhile, in colorectal cancer, exosomal lncRNA *SNHG10* upregulates the NK cell negative receptor INHBC and disrupts the function of these immune effectors.^95^

However, some exosomes can inhibit tumor metastasis. This is the case of exosomes derived from bone marrow stromal cells (BMSC), which may transport *miR‐506*, an inhibitor of tumor growth and metastatic features of human glioma cells.^96^ Additional research demonstrates that the use of BMSC‐derived exosomes loaded with *miR‐512‐5p* not only inhibits glioblastoma growth in vitro but also in vivo, prolonging survival in mice by reducing the expression of *JAG1*,^97^ a cell surface ligand that plays a crucial role in tumor development and malignant glioma progression.^98^, ^99^, ^100^

## 5 CROSSTALK BETWEEN EXOSOMES AND AUTOPHAGY

Even though exosome biogenesis and autophagic degradation seem distant at first, they both share the need for lipid mobilization, membrane reorganization, and vesicle transport. Thus, it is not surprising that a direct link has been described between them, as they can even share common cellular structures. In macroautophagy, the autophagosome fuses with the lysosome to form an autolysosome for the degradation of autophagosomal cargo. However, in a previous step, the autophagosome may combine with a multivesicular body (MVB) to form an organelle called “amphisome.” This structure, rather than fusing with the lysosome, might merge with the plasma membrane, releasing exosomes into the extracellular medium.^10^ This route is, therefore, an excellent example of direct crosstalk between exosomes and autophagy. However, it is important to clarify that amphisomes are not only an alternative way for cells to secrete exosomes, but also a mechanism to release unwanted double‐stranded DNA (dsDNA) and histones through an exosome‐independent mechanism.^8^

**FIGURE 2:**
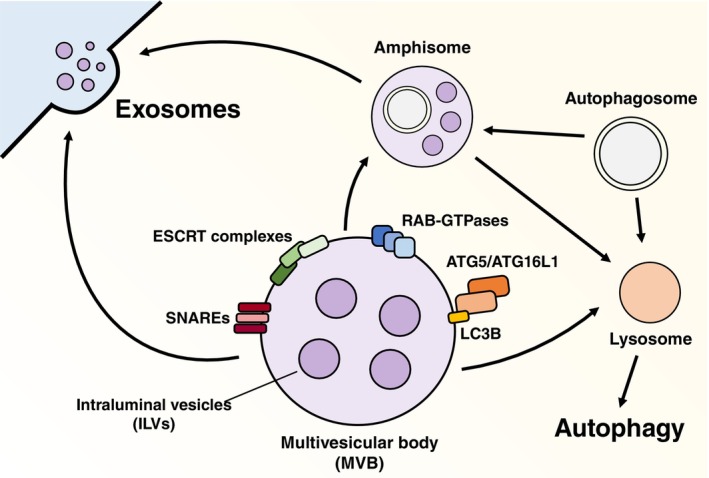
Crosstalk between exosomes and autophagy. Though distinct cellular pathways, the biogenesis of exosomes and the autophagic response share common structures (like the multivesicular body, MVB) and effectors (like the SNARE and RAB‐GTPase protein families, as well as the ESCRT complexes and autophagy‐related [ATG] proteins).

Common mediators of exosome secretion and autophagy have also been described^101^ (Figure 2). The endosomal sorting complexes required for transport (ESCRT), for example, are a group of protein complexes that play a crucial role in various cellular processes, including the sorting of ubiquitinated membrane proteins for degradation, cytokinesis, and MVB biogenesis. It consists of four complexes (ESCRT‐0, ‐I, ‐II, and ‐III) and associated proteins (like VPS4 or ALIX).^102^ The ESCRT machinery participates in the budding of the endosomal limiting membrane to generate intraluminal vesicles (ILVs) within MVBs. When MVBs fuse with the cell membrane, ILVs are released as exosomes into the extracellular space. Additionally, the ESCRT machinery is responsible for the selective packaging of cargo into exosomes, ensuring the highly selective and tightly regulated nature of exosomal cargo.^103^ The ESCRT complexes also play a significant role in autophagy,^104^ particularly in the formation of autophagosomes, as they are involved in the sorting of ubiquitinated cargo into ILVs within MVBs, a process that is essential for autophagosome maturation and cargo degradation. ALIX is one of these ESCRT‐related proteins at the crosstalk between exosomal release and autophagy. Specifically, its interaction with the ATG12‐ATG3 autophagy complex^105^ controls processes like late endosome distribution, viral budding, and exosome biogenesis. Furthermore, ALIX is sequestered by ATG12‐ATG3 when ATG5 is repressed, preventing the reparation of lysosomes and increasing the secretion of extracellular vesicles.^106^

RAB‐GTPases also play a role in autophagic flux and exosome secretion. RAB7 is required for the transfer of cargo from MVBs to the lysosome, where lysosomal enzymes will degrade it.^107^ It may also participate in the regulation of the trafficking of MVBs, mediating the translocation of MVBs along microtubules to the cell periphery, and in the docking and fusion events that lead to exosome release.^108^ In autophagy, RAB7 regulates the maturation and trafficking of autophagosomes.^109^ Another RAB‐GTPase, RAB11, has been shown to promote the docking and fusion of MVBs too, contributing to exosome secretion, and it participates in the recycling of cargo from the endocytic recycling compartment to the plasma membrane.^110^, ^111^ Studies have shown that RAB11 also regulates the fusion of autophagosomes containing ANXA2 (a protein that can be exported by exosomes under IFN‐γ stimulation) and MVBs to form amphisomes.^112^ As RAB‐GTPases, the soluble N‐ethylmaleimide‐sensitive factor attachment protein receptors (SNARE) family participates in the fusion events of MVBs with the plasma membrane.^113^ Still, it also mediates autophagosome–lysosome fusion.^114^ MAP1LC3B (Microtubule‐associated protein 1A/1B‐light chain 3B), a protein that is necessary to recruit specific cargo to the autophagosome during autophagy,^115^ is also involved in the packaging of cytosolic cargoes into extracellular vesicles for secretion outside the cell.^116^ This process has been described as a secretory autophagy pathway termed LC3‐dependent EV loading and secretion (LDELS), which is interestingly dependent on the ESCRT machinery and another small GTPase: RAB27A.^117^

However, the complex relationship between autophagy and exosome biogenesis and release goes beyond sharing common effectors. In fact, they can directly regulate each other. Specifically, stressful stimuli and pathological conditions can paracrinally affect autophagy through the release of exosomes; conversely, autophagy levels may modulate the formation and release of exosomes, demonstrating that these pathways are cross‐regulated.^118^ For example, Fader et al. showed that autophagy inducers such as starvation or rapamycin caused a reduction in exosome release, suggesting that under autophagy‐inducing conditions, MVBs are directed to the autophagic pathway,^119^ hindering the production and liberation of exosomes. However, some autophagy effectors (like ATG5 and ATG16L1) favor exosome release after preventing the acidification and subsequent lysosomal degradation of MVBs by disassociating the V‐ATPase from the MVB membrane.^120^ As for the effect of exosomes on autophagy, the content of these extracellular vesicles has been shown to interact with the PI3K/AKT/mTOR or AMPK/mTOR pathways,^121^, ^122^ which are key regulators of autophagy (being AMPK one of their main activators and mTOR or AKT prominent inhibitors).^123^, ^124^ For example, Song et al. found that exosomal *miR‐7‐5p* targets *EGFR* in bronchial epithelial cells, preventing the phosphorylation of AKT and MTOR and thus activating autophagy.^125^ Besides, Ren et al. showed that exosomes from adipose‐derived stem cells inhibited autophagy through the downregulation of p‐AMPK, restoring ovarian function in premature ovarian failure (POF).^126^ Another way in which exosomes can regulate autophagy is by acting on Beclin‐1. Beclin‐1 is encoded by *BECN1*, which is the target gene of *miR‐30a* in cancer, hepatic fibrosis, or diabetic cataracts.^127^, ^128^, ^129^ Accordingly, exosomal *miR‐30a* can reduce autophagy levels in oral squamous cell carcinoma by targeting *BECN1*.^130^ Moreover, exosomal *miR‐376a‐3p*, isolated from the peripheral blood of patients with Crohn Disease (CD), can target the autophagy‐related protease *ATG4C*, which has been identified as an associated risk factor for CD.^131^

## 6 THE EXOSOMES‐AUTOPHAGY CONNECTION IN CANCER

Autophagy plays a dual role in cancer progression: it can function as a tumor suppressor by inhibiting malignant transformation at initial stages or inducing cell death, while it also has a fundamental role in assisting cancer cells to survive at advanced stages.^132^ Additionally, autophagy can serve both as a mechanism for chemotherapy resistance acquisition or as an enhancer of certain anticancer therapies.^133^, ^134^ Given this complex role of autophagy in cancer and the interplay between this pathway and exosomes, it is not surprising that a growing number of studies are describing heterogeneous changes in the autophagic response and exosomal biogenesis in the context of this pathology.

In some cases, both autophagy and exosome release are activated in cancer cells by common pathways. In tumors, hypoxia can induce autophagy, which prevents inflammation and cell death,^135^ and many studies have demonstrated that cancer cells secrete more exosomes under hypoxic conditions, with their cargo being altered by this type of stress.^136^, ^137^ In this regard, the hypoxia‐inducible factor 1 alpha (HIF‐1α), a key regulator of the cellular response to low oxygen levels and a well‐known autophagy activator, is involved in the packaging and delivery of exosomes, and its presence in exosomes has been associated with pro‐metastatic effects.^138^ Interestingly, HIF‐1α can control the transcription of several miRNAs that are enriched in exosomes derived from hypoxic cells, such as *mir‐23a*, which targets BCL2 interacting protein 3‐like (*BNIP3L*), a crucial mitophagy receptor.^139^ Autophagy and exosome release can also be upregulated in response to other cellular stresses in cancer cells, such as the unfolding protein response (UPR) and the endoplasmic reticulum (ER) stress.^140^ For example, the downregulation of PKR‐like ER kinase (PERK) and inositol‐requiring protein 1 (IRE1), two key effectors of the UPR pathway that also crosstalk with autophagy,^141^ abrogates the increase in exosome release.^142^

On the other hand, specific exosomal miRNAs that are potential tumor promoters can also modulate autophagic‐related responses. For example, exosomes released by breast cancer cells carry *miR‐1910‐3p*, which promotes autophagy, proliferation, and metastasis.^37^ Moreover, Guo et al. showed that lncRNA *H19* is overexpressed in exosomes derived from tumor‐associated macrophages, and that their administration to bladder cancer cells promotes autophagy by suppressing the interaction between ULK1 and NEDD4L.^143^ Wang et al. also provided evidence that exosomes secreted by gastric cancer cells overexpressing TOB1 can induce autophagy in other tumor cells by decreasing the activation of the AKT/mTOR signaling pathway.^144^ However, tumoral exosomes can also repress autophagy to promote invasiveness and accelerated metastatic progression, as shown by exosomal *miR‐1268a* in melanoma.^145^

Interestingly, several exosomal miRNAs derived from cancer cells have been identified as either drug resistance or enhancer factors through the modulation of autophagy in different cancers, becoming potential therapeutic targets. In non‐small cell lung cancer (NSCLC), the cisplatin‐induced release of exosomes enriched with *miR‐425‐3p* can enhance autophagy by targeting AKT, ultimately leading to chemoresistance and tumor growth, and contributing to the progression of lung cancer.^146^ Furthermore, drug‐resistant osteosarcoma cells secrete exosomes with higher levels of exosomal *miR‐331‐3p* that can be absorbed by regular osteosarcoma cells, inducing autophagy and allowing them to acquire drug resistance.^147^ By contrast, Kulkarni et al. demonstrated that exosomal‐mediated delivery of *miR‐30a* leads to decreased autophagic activity by modulating the expression of Beclin‐1, sensitizing cisplatin‐resistant oral squamous carcinoma cells.^130^ Other exosomal cargo may indirectly induce autophagy in cancer cells by targeting specific miRNAs. This is the case of exosomal circRNA‐plasmacytoma variant translocation 1 (*circ‐PVT1*), which downregulates *miR‐30a‐5p*, intensifying gastric cancer cells cisplatin‐resistance through autophagy modulation,^148^ while exosomal lncRNA *Linc00969*, which is overexpressed in trastuzumab‐resistant patients with breast cancer, can disseminate drug resistance by inducing autophagy.^149^ Zhu and collaborators have demonstrated that *lnc‐FAL1*, a lncRNA that promotes oxaliplatin chemoresistance in colorectal cancer (CRC), is mainly derived from exosomes secreted by cancer‐associated fibroblasts (CAFs), inhibiting oxaliplatin‐induced autophagy by acting as a scaffold for the interaction between Beclin‐1 and TRIM3 (promoting TRIM3‐dependent Beclin‐1 polyubiquitination and degradation).^150^ Another study showed that *circ_0091741* delivered by gastric cancer cell‐derived exosomes induced autophagy and oxaliplatin resistance in gastric cancer cells by regulating the Wnt/β‐catenin axis.^151^ Exosomes derived from hepatitis B virus (HBV)‐associated liver cancer cells promote chemoresistance by modulating the chaperone‐mediated autophagy pathway.^152^ Lastly, treatments based on the co‐administration of tyrosine kinase inhibitors (TKIs, such as gefitinib) with chemotherapy agents fail to improve overall survival in advanced non‐small cell lung cancer patients, as exosomes released by gefitinib‐treated PC9 cells activate autophagy and reduce cisplatin‐induced apoptosis.^153^

Finally, the crosstalk between exosomes and autophagy can also modify other cell types in the tumor microenvironment. Exosomes containing *KRAS* ^*G12D*^, the most common mutation of *KRAS* in pancreatic ductal adenocarcinoma (PDAC), can be released by cancer cells after autophagy‐dependent ferroptosis, transferring the mutation to macrophages and switching them to an M2‐like phenotype.^154^ However, when the same ferroptosis‐dependent exosome release and macrophagic uptake occur in breast cancer, the expression of up to 84 autophagy‐related genes is altered, resulting in suppressed M2 macrophage polarization and the inhibition of breast cancer cell migration and invasion.^155^ Additionally, a recent study has shown that tumor‐derived exosomes, which are enriched with five autophagy‐related proteins (LC3B, CK2, RAB7A, SQSTM1, and UB) and are secreted by breast cancer cells shown the ability to activate autophagy in normal epithelial breast cells,^156^ resulting in the release of cancer cell growth‐promoting factors, establishing a tumor microenvironment that is suitable for the proliferation of cancer cells.^157^

## 7 FINAL REMARKS

**FIGURE 3:**
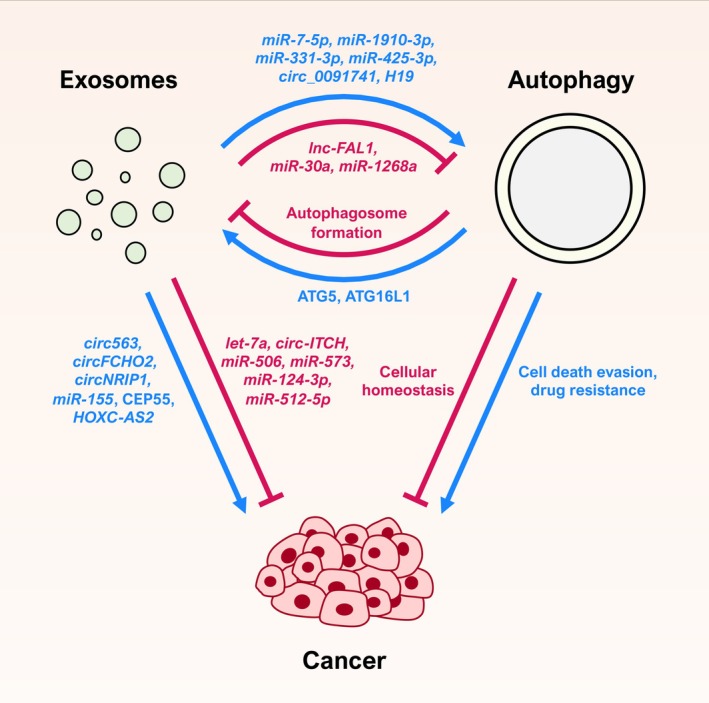
The exosomes‐autophagy‐cancer triad. The interplay between exosomes, autophagy, and cancer is a complex one where both the extracellular vesicles and the cellular recycling process show dual roles when regulating each other or the disease, depending on the context. Some of the modulators described throughout the text are depicted in the diagram.

The crosstalk between autophagy and exosomes is relevant in cancer, but also complex (Figure 3), due to the dualism that is present in this cellular crossroad at different levels. As shown throughout this review, autophagy can repress or promote tumor progression depending on the context, and the role of exosomes in cancer depends on the cargo they transport and deliver. Even more, their mutual effect is also dual, as the autophagic response can facilitate or hamper the production and release of exosomes, while these extracellular vesicles can trigger the activation or repression of autophagy in receiving cells. This dualism implies that any experimental intervention on autophagy or exosome biogenesis should take into consideration possible alterations on the other. But it also means that these two processes can be concurrently targeted when developing new therapeutic strategies. However, the possibility of modulating both exosome release and autophagy to correct their dysregulation in cancer remains unexplored.

It is particularly intriguing that these pathways share common components in their molecular machinery. Given the fact that some pathologies show simultaneous dysregulation of both processes, exploring ways to correct these alterations at once would be of much interest. In this regard, molecules acting in both pathways (like the ESCRT components or RAB‐GTPase proteins) may become potential targets for concomitant autophagy/exosome modulation, aiming to either enhance or suppress their activities. Besides these common effectors, autophagy and exosomes also have a shared need: lipid and/or membrane supply (specifically with macroautophagy, a major type of autophagic response characterized by the formation of double‐membrane vesicles termed autophagosomes). Thus, new studies aimed at better understanding lipid dynamics during autophagosome and exosome formation are needed, exploring the possibility of autophagy/exosome modulation by providing or limiting access to these biomolecules.

## AUTHOR CONTRIBUTIONS

**María Guerra‐Andrés:** Writing – original draft; conceptualization. **Álvaro F. Fernández:** Conceptualization; writing – original draft; writing – review and editing; supervision. **Tania Fontanil:** Conceptualization; writing – original draft; writing – review and editing; supervision.

## FUNDING INFORMATION

The study of Álvaro F. Fernández is supported by grants CNS2024‐154954 and PID2021‐127534OB‐I00, funded by MICIU/AEI/10.13039/501100011033 and by ERDF/EU, as well as a Beatriz Galindo grant from the Ministerio de Universidades of Spain (MCIU‐20‐BG20/00030). The study of María Guerra‐Andrés is supported by a predoctoral grant from the University of Oviedo (PAPI‐22‐PF‐21).

## CONFLICT OF INTEREST STATEMENT

The authors declare that they have no known competing financial interests or personal relationships that could have appeared to influence the work reported in this paper.
